# Age-related differences of serum total testosterone and psychological factors in patients with erectile dysfunction

**DOI:** 10.3389/fendo.2025.1615402

**Published:** 2025-10-30

**Authors:** Zhaoxu Yang, Chencong Lu, Qing Wang, Tao Liu, Yan Xu, Xinfei Huang, Jianhuai Chen

**Affiliations:** ^1^ Department of Andrology, Jiangsu Province Hospital of Chinese Medicine, Affiliated Hospital of Nanjing University of Chinese Medicine, Nanjing, China; ^2^ Department of Surgery, Hai’an Hospital of Traditional Chinese Medicine, Nantong, China; ^3^ Department of Andrology, Nanjing Hospital of Traditional Chinese Medicine, Nanjing Hospital of Chinese Medicine Affiliated to Nanjing University of Chinese Medicine, Nanjing, Jiangsu, China

**Keywords:** erectile dysfunction, age, psychological factors, anxiety, depression, testosterone

## Abstract

**Objective:**

This study aimed to explore the differences of the psychological states and total testosterone (TT) in patients with erectile dysfunction (ED) across various age groups.

**Methods:**

A total of 1411 ED patients were enrolled from the Department of Andrology, Jiangsu Province Hospital of Chinese Medicine, Affiliated Hospital of Nanjing University of Chinese Medicine from September 2018 to September 2021. The SCL-90 was used to evaluate the psychological condition of patients while the 5-item international index of erectile function (IIEF-5) questionnaire was applied to estimate the severity of ED. The serum TT level of patients was also measured. ED patients were divided into three groups (group A: 20–30 years old; group B: 31–40 years old; group C: 41–50 years old). In addition, patients in each group were divided into three groups including mild group (12<IIEF-5<21), moderate group (8<IIEF-5<11), and severe group (IIEF-5<7). The level of TT and SCL-90 scores were compared between groups. Finally, relationships between SCL-90, STAI scores and TT were explored.

**Results:**

(1) Differences of TT between groups with different ages: The TT level of group A was higher than that of group B and C (P<0.05). (2) Differences of TT between patients with different ED severity in each age group: The TT level of severe ED patients was lower than that of mild ED patients in group B (P<0.05) while the TT level of severe ED patients was lower than that of mild and moderate ED patients in group C (P<0.05). (3) Differences of SCL-90 and STAI scores between patients with different severity in each age group: The factor scores of anxiety, psychoticism, obsession, interpersonal relationship, depression and total scores of SCL-90 of group A and B were higher than those of group C (P<0.05). (4) Differences of SCL-90 and STAI scores between patients with different severity in each age group: 1) The factor scores of hostility, anxiety, phobia, paranoid, psychoticism, obsession, depression and total scores of SCL-90 of mild ED patients were lower than those of moderate and severe ED patients in group B (P<0.05); 2) The factor scores of anxiety and other of SCL-90, as well as the state anxiety scores of STAI of mild ED patients were lower than those of severe ED patients in group C (P<0.05). (5) The level of TT was positively related to IIEF-5 scores of ED patients (*r* = 0.06; *P* = 0.02). Both SCL-90 (*r* = -0.08; *P* < 0.01) and STAI (*r* = -0.06; *P* = 0.04) scores were negatively associated with IIEF-5 scores of ED patients. STAI scores were positively related to SCL-90 scores of ED patients (*r* = 0.64; *P* < 0.01).

**Conclusion:**

Age-stratified results demonstrate a pronounced differential impact of TT and psychological factors on ED. Compared with younger patients, serum TT has more significant effects on elderly patients with ED. For young ED patients, the influences of psychological factors are significantly higher than that of elderly patients, and young patients show more severe anxiety and depression.

## 1 Introduction

Erectile dysfunction (ED), a common male disease, is considered as a complex and multifactorial disease, which seriously affects the quality of life of patients (1, 2). At present, ED is divided into organic, psychological and mixed according to its causes (2, 3). ED is affected by various factors, which have different effects on patients of different ages, such as vascular, neurological, psychological, and endocrine hormones (4). Epidemiological evidences from multinational cohort studies consistently demonstrate a significantly higher prevalence of ED in elderly males (>40 years) compared to younger populations. The prevalence of ED in elderly males reaches 37% (5–7). The influencing factors for ED were focused on elderly patients over 40 years old, while the research on young ED patients was ignored (8).

For young patients with ED, the prevalence of common ED related complications is much lower than that of older patients (9). Young ED patients (≤40 years) exhibit significantly lower prevalence rates of comorbidities: hypogonadism (10.8% vs 38.7%), hypertension (15.6% vs 29%), cardiovascular disease (3.3% vs 16%) compared to older their older patients (≥40 years) (9–11). However, the number of young patients under the age of 40 seeking treatment for ED is gradually increasing (12). The ED consultation rates among men under 40 years, rise from 5% of total cases in 2010 to 15% in 2015 (8). Testosterone mediates penile vasodilation via nitric oxide synthase activation (13), however, the correlation between total testosterone (TT) levels and ED remains inconclusive. This discrepancy may reflect the multifactorial nature of ED pathogenesis across different age groups. The characteristics of the overlap of testosterone deficiency symptoms with other diseases make the diagnosis of testosterone deficiency and its related comorbidities prone to missed diagnosis or overtreatment (14–16). TT plays a key role in male sexual response, and symptoms associated with low testosterone such as hypogonadism, are also important risk factors for ED (17–20). However, the age-related TT decline (21) contradicts increased ED incidence in younger adults (22), suggesting multifactorial pathophysiology.

In addition, the psychological factors are key risk factors for the occurrence and development of ED (23–25). A retrospective study involving 3500 patients aged 18–48 showed that depression and anxiety were important predictors of young ED patients (26). The incidence of anxiety and depression symptoms in ED patients are significantly higher than that in healthy people (17.1% vs 12.9%) (27). These evidences indicate that there may be a bidirectional effect between psychological factors and ED (28–31). Given the age-dependent roles of testosterone deficiency and psychological distress in ED pathogenesis, in this cross-sectional study, TT levels and psychological assessments were collected from 1,411 ED patients stratified by age. The objective was to elucidate the differential contributions of TT and psychogenic factors to ED across distinct age groups.

## 2 Materials and methods

### 2.1 Participants

This study was approved by the ethical committee of Jiangsu Province Hospital of Chinese Medicine, Affiliated Hospital of Nanjing University of Chinese Medicine. Moreover, written informed consents were obtained from all patients. A total of 1411 ED patients were enrolled from the Department of Andrology, Jiangsu Province Hospital of Chinese Medicine, Affiliated Hospital of Nanjing University of Chinese Medicine from September 2018 to September 2021.

The patients enrolled in this study were aged 20–50 years, who had a regular sexual partner and regular sexual life for more than 6 months. Those with organic abnormalities of penis and testis, combined with other types of sexual dysfunction, sexual partners with sexual dysfunction, previous psychiatric history, organic mental disorders or mental disorders were excluded.

### 2.2 Instruments

#### 2.2.1 Serum TT

Fasting venous blood was drawn from the subjects at 8:00 in the morning, and the TT level was detected by chemiluminescence assay (normal value range: 248.00~835.00ng/dL).

#### 2.2.2 Symptom checklist 90

SCL-90 is a self-reported symptom scale, which is often used to evaluate psychopathological symptoms. The scale includes 10 main symptom dimensions: somatization, obsession, interpersonal relationship, depression, anxiety, hostility, phobia, paranoid, psychoticism and other. In this study, each question has a score of 0-4. The lower the score, the better the psychological health condition.

#### 2.2.3 State-trait anxiety inventory

STAI is a self-report questionnaire consisting of 2 scales with 20 questions. Questions 1–20 are state anxiety scale, which is used to assess immediate or recent experiences and feelings. Questions 21–40 are trait anxiety scale, which is used to assess people’s regular emotional experience. Each question has a score of 1-4.

### 2.3 Statistical analysis

ED patients were divided into three groups (group A: 20–30 years old, n=704; group B: 31–40 years old, n=457; group C: 41–50 years old, n=250). The TT level and psychological evaluation data were analyzed by ANOVA and Tukey’s *post hoc*-test for pairwise comparisons (LSD test) in each group. ED patients in each age group were divided into three groups including mild group (12<IIEF-5<21), moderate group (8<IIEF-5<11), and severe group (IIEF-5<7). The data of TT level and psychological evaluation of ED patients with different levels of severity were analyzed by ANOVA and Tukey’s *post hoc*-test for pairwise comparisons (LSD test) in different age groups. Finally, relationships between SCL-90, STAI scores and TT were explored. All analyses were carried out with SPSS 25 statistical package (IBM Corporation, Armonk, NY, USA), and P<0.05 was considered statistically significant.

## 3 Result

### 3.1 Comparison of TT in each age group

Compared with 704 patients in group A, 457 patients in group B and 250 patients in group C had significantly lower TT levels. [A (447.35 ± 127.46) ng/dL VS B (430.62 ± 116.14) ng/dL, C (425.72 ± 93.47) ng/dL)] (P<0.05).

### 3.2 Comparison of TT in patients with different ED severity in each age group

#### 3.2.1 Group A

There was no statistically significant difference in the TT level between 443 patients with mild ED and 241 patients with moderate ED and 20 patients with severe ED (P>0.05) (**Table 1**).

**Table 1:** TT data of ED patients in group A.

Severity	Mean ± standard deviation (ng/dL)	F value	Mild	T value	P value	Moderate	T value	P value
Mild	448.893 ± 130.349	0.784	–	–	–	–	–	–
Moderate	447.426 ± 125.045		1.467	0.144	0.886	–	–	–
Severe	412.406 ± 83.945		36.487	1.252	0.211	35.020	1.180	0.238

#### 3.2.2 Group B

Compared with that in 224 patients with mild ED, the level of TT was significantly declined in 40 patients with severe ED (P<0.05). There was no statistically significant difference in the TT level between 193 patients with moderate ED and patients with mild or severe ED (P>0.05) (**Table 2**).

**Table 2:** TT data of ED patients in group B.

Severity	Mean ± standard deviation(ng/dL)	F value	mild	T value	P value	moderate	T value	P value
Mild	439.269 ± 110.688	3.092	–	–	–	–	–	–
Moderate	428.972 ± 127.420		10.297	0.907	0.365	–	–	–
Severe	390.189 ± 174.939		49.080	2.473	0.014	38.783	1.931	0.054

#### 3.2.3 Group C

Compared with that in 128 patients with mild ED and 89 patients with moderate ED, the level of TT was significantly declined in 33 patients with severe ED (P<0.05). No statistically significant difference was observed in the TT level among mild and moderate patients (P>0.05) (**Table 3**).

**Table 3:** TT data of ED patients in group C.

Severity	Mean ± standard deviation (ng/dL)	F value	Mild	T value	P value	Moderate	T value	P value
Mild	430.853 ± 88.232	2.347	–	–	–	–	–	–
Moderate	430.451 ± 100.305		0.402	0.031	0.975	–	–	–
Severe	393.059 ± 90.303		37.795	2.082	0.038	37.393	1.973	0.05

### 3.3 Comparison of SCL-90 and STAI scores in each age group

The factor scores of anxiety, psychoticism, obsession, interpersonal relationship, depression and total scores of SCL-90 of group A and B were higher than those of group C (P<0.05). The factor scores of trait anxiety, state anxiety and total scores of STAI of group A and B were higher than those of group C (P<0.05) (**Table 4**).

**Table 4:** SCL-90 and STAI scores in each age group.

Factors	Group	Factor Scores	F value	Group A	T value	P value	Group B	T value	P value
Hostility	Group A	3.59 ± 3.226	2.744	–	–	–	–	–	–
	Group B	4.07 ± 4.064		-0.481	-2.217	0.027	–	–	–
	Group C	3.59 ± 3.746		-0.001	-0.004	0.997	0.48	1.690	0.091
Anxiety	Group A	6.54 ± 5.281	7.559	–	–	–	–	–	–
	Group B	6.32 ± 5.801		0.218	0.669	0.504	–	–	–
	Group C	5 ± 5.128		1.536	3.840	<0.0001	1.318	3.087	0.002
Psychoticism	Group A	6.59 ± 5.261	4.04	–	–	–	–	–	–
	Group B	6.57 ± 5.707		0.02	0.062	0.951	–	–	–
	Group C	5.52 ± 4.991		1.071	2.711	0.007	1.051	2.491	0.013
Phobia	Group A	2.32 ± 2.853	3.519	–	–	–	–	–	–
	Group B	2.19 ± 2.906		0.138	0.802	0.424	–	–	–
	Group C	1.76 ± 2.836		0.560	2.654	0.008	0.422	1.867	0.062
Paranoid	Group A	3.25 ± 3.091	2.889	–	–	–	–	–	–
	Group B	3.51 ± 3.524		-0.263	-1.342	0.181	–	–	–
	Group C	2.9 ± 3.268		0.351	1.456	0.144	.614	2.389	0.017
Other	Group A	4.2 ± 3.904	2.187	–	–	–	–	–	–
	Group B	4.7 ± 4.419		-0.506	-2.040	0.042	–	–	–
	Group C	4.26 ± 4.228		-0.061	-0.201	0.841	0.445	1.369	0.172
Obsession	Group A	9.58 ± 5.799	6.554	–	–	–	–	–	–
	Group B	9.38 ± 6.419		0.207	0.575	0.565	–	–	–
	Group C	8.01 ± 5.731		1.576	3.574	<0.0001	1.368	2.898	0.004
Somatization	Group A	5.47 ± 5.19	0.492	–	–	–	–	–	–
	Group B	5.79 ± 6.038		-0.325	-0.982	0.327	–	–	–
	Group C	5.54 ± 5.417		-0.069	-0.170	0.866	0.256	0.590	0.555
Interpersonal Relationship	Group A	7.09 ± 5.401	4.246	–	–	–	–	–	–
	Group B	7.22 ± 5.927		-0.129	-0.385	0.701	–	–	–
	Group C	6.02 ± 5.394		1.072	2.615	0.009	1.201	2.736	0.006
Depression	Group A	9.3 ± 7.668	3.651	–	–	–	–	–	–
	Group B	9.44 ± 8.515		-0.146	-0.306	0.76	–	–	–
	Group C	7.87 ± 7.639		1.430	2.444	0.015	1.576	2.522	0.012
Total scores of SCL-90	Group A	57.97 ± 40.475	3.671	–	–	–	–	–	–
	Group B	59.28 ± 46.878		-1.314	-0.509	0.611	–	–	–
	Group C	50.52 ± 42.101		7.449	2.357	0.019	8.762	2.595	0.01
Trait Anxiety	Group A	45.6 ± 8.782	20.471	–	–	–	–	–	–
	Group B	45.04 ± 9.426		0.559	1.004	0.316	–	–	–
	Group C	41.3 ± 10.198		4.300	6.305	<0.0001	3.741	5.132	<0.0001
State Anxiety	Group A	45.04 ± 10.11	17.045	–	–	–	–	–	–
	Group B	44.1 ± 10.14		0.937	1.544	0.123	–	–	–
	Group C	40.71 ± 9.886		4.328	5.825	<0.0001	3.391	4.271	<0.0001
Total scores of STAI	Group A	90.39 ± 19.009	17.968	–	–	–	–	–	–
	Group B	88.81 ± 19.681		1.574	1.355	0.176	–	–	–
	Group C	81.89 ± 19.66		8.496	5.966	<0.0001	6.922	4.548	<0.0001

### 3.4 Differences of SCL-90 and STAI scores between patients with different ED severity in each age group

#### 3.4.1 Group A

There were no significant differences in SCL-90 and STAI scores of mild, moderate and severe ED patients (P>0.05).

#### 3.4.2 Group B

There were no significant differences in SCL-90 and STAI scores of moderate and severe ED patients (P>0.05). The factor scores of hostility, anxiety, phobia, paranoid, psychoticism, obsession, depression and total scores of SCL-90 of mild ED patients were lower than those of moderate and severe ED patients (P<0.05). The factor scores of trait anxiety, state anxiety and total scores of STAI of mild ED patients were lower than those of moderate and severe ED patients (P<0.05) (**Table 5**).

**Table 5:** SCL-90 and STAI scores between patients with different severity in group B.

Factors	Group	Factor Scores	F value	Mild	T value	P value	Moderate	T value	P value
Hostility	Mild	3.54 ± 3.613	4.476	–	–	–	–	–	–
	Moderate	4.44 ± 4.285		-0.891	-2.250	0.025	–	–	–
	Severe	5.28 ± 4.935		-1.730	-2.500	0.013	-0.84	-1.198	0.231
Anxiety	Mild	5.45 ± 5.02	5.782	–	–	–	–	–	–
	Moderate	6.96 ± 6.22		-1.517	-2.690	0.007	–	–	–
	Severe	8.13 ± 7.013		-2.679	-2.717	0.007	-1.161	-1.164	0.245
Psychoticism	Mild	5.55 ± 5.095	8.359	–	–	–	–	–	–
	Moderate	7.28 ± 5.711		-1.726	-3.127	0.002	–	–	–
	Severe	8.8 ± 7.637		-3.246	-3.367	0.001	-1.52	-1.557	0.12
Phobia	Mild	1.83 ± 2.522	3.712	–	–	–	–	–	–
	Moderate	2.44 ± 3.227		-0.606	-2.134	0.033	–	–	–
	Severe	2.93 ± 3.083		-1.090	-2.198	0.028	-0.485	-0.966	0.335
Paranoid	Mild	3 ± 3.093	5.586	–	–	–	–	–	–
	Moderate	3.88 ± 3.712		-0.876	-2.554	0.011	–	–	–
	Severe	4.65 ± 4.383		-1.650	-2.755	0.006	-0.774	-1.277	0.202
Other	Mild	4.41 ± 4.404	0.977	–	–	–	–	–	–
	Moderate	4.97 ± 4.349		-0.563	-1.297	0.195	–	–	–
	Severe	5.05 ± 4.83		-0.639	-0.842	0.4	-0.076	-0.099	0.921
Obsession	Mild	8.33 ± 5.748	6.376	–	–	–	–	–	–
	Moderate	10.2 ± 6.554		-1.862	-2.989	0.003	–	–	–
	Severe	11.25 ± 8.258		-2.915	-2.677	0.008	-1.053	-0.956	0.34
Somatization	Mild	5.46 ± 5.665	1.739	–	–	–	–	–	–
	Moderate	5.85 ± 5.936		-0.4	-0.676	0.5	–	–	–
	Severe	7.38 ± 8.129		-1.92	-1.855	0.064	-1.52	-1.452	0.147
Interpersonal Relationship	Mild	6.21 ± 5.175	8.957	–	–	–	–	–	–
	Moderate	7.81 ± 6.181		-1.594	-2.787	0.006	–	–	–
	Severe	10.03 ± 7.343		-3.811	-3.811	0	-2.217	-2.191	0.029
Depression	Mild	8.08 ± 7.599	6.615	–	–	–	–	–	–
	Moderate	10.43 ± 8.674		-2.350	-2.845	0.005	–	–	–
	Severe	12.33 ± 11.097		-4.245	-2.940	0.003	-1.895	-1.297	0.195
Total scores of SCL-90	Mild	52 ± 41.559	6.458	–	–	–	–	–	–
	Moderate	64.31 ± 48.123		-12.315	-2.707	0.007	–	–	–
	Severe	75.83 ± 60.889		-23.829	-2.997	0.003	-11.514	-1.431	0.153
Trait Anxiety	Mild	43.72 ± 8.903	4.725	–	–	–	–	–	–
	Moderate	46.05 ± 8.929		-2.330	-2.530	0.012	–	–	–
	Severe	47.5 ± 13.095		-3.778	-2.354	0.019	-1.448	-0.891	0.373
State Anxiety	Mild	43.04 ± 9.988	2.887	–	–	–	–	–	–
	Moderate	44.83 ± 9.31		-1.793	-1.804	0.072	–	–	–
	Severe	46.53 ± 13.767		-3.485	-2.010	0.045	-1.692	-0.964	0.336
Total scores of STAI	Mild	86.47 ± 18.772	3.708	–	–	–	–	–	–
	Moderate	90.46 ± 18.816		-3.987	-2.075	0.039	–	–	–
	Severe	94.03 ± 26.418		-7.556	-2.250	0.025	-3.569	-1.050	0.294

#### 3.4.3 Group C

There were no significant differences in SCL-90 and STAI scores of mild and moderate ED patients (P>0.05). The factor scores of anxiety and other of SCL-90, as well as the state anxiety scores of STAI of mild ED patients were lower than those of severe ED patients (P<0.05). Furthermore, the factor scores of interpersonal relationship, depression and total scores of SCL-90 of moderate ED patients were higher than those of severe ED patients (P<0.05) (**Table 6**).

**Table 6:** SCL-90 and STAI scores between patients with different severity in group C.

Factors	Group	Factor Scores	F value	Mild	T value	P value	Moderate	T value	P value
Hostility	Mild	3.52 ± 3.578	1.775	–	–	–	–	–	–
	Moderate	3.28 ± 3.451		0.243	0.472	0.638	–	–	–
	Severe	4.7 ± 4.908		-1.174	-1.610	0.109	-1.416	-1.861	0.064
Anxiety	Mild	4.98 ± 5.153	3.532	–	–	–	–	–	–
	Moderate	4.28 ± 3.811		0.703	1.003	0.316	–	–	–
	Severe	7.03 ± 7.325		-2.046	-2.065	0.04	-2.749	-2.656	0.008
Psychoticism	Mild	5.55 ± 4.717	1.279	–	–	–	–	–	–
	Moderate	5.04 ± 4.436		0.502	0.730	0.466	–	–	–
	Severe	6.67 ± 7.03		-1.12	-1.151	0.251	-1.622	-1.596	0.112
Phobia	Mild	1.77 ± 2.719	0.646	–	–	–	–	–	–
	Moderate	1.58 ± 2.178		0.181	0.462	0.644	–	–	–
	Severe	2.24 ± 4.458		-0.477	-0.859	0.391	-0.658	-1.136	0.257
Paranoid	Mild	2.91 ± 3.153	1.077	–	–	–	–	–	–
	Moderate	2.63 ± 2.917		0.277	0.614	0.539	–	–	–
	Severe	3.61 ± 4.415		-0.7	-1.097	0.274	-0.977	-1.467	0.144
Other	Mild	4.13 ± 4.016	4.13	–	–	–	–	–	–
	Moderate	3.74 ± 3.764		0.391	0.679	0.498	–	–	–
	Severe	6.15 ± 5.624		-2.019	-2.477	0.014	-2.410	-2.832	0.005
Obsession	Mild	7.98 ± 5.848	1.585	–	–	–	–	–	–
	Moderate	7.47 ± 5.419		0.512	0.649	0.517	–	–	–
	Severe	9.55 ± 5.985		-1.561	-1.399	0.163	-2.074	-1.780	0.076
Somatization	Mild	5.31 ± 5.019	1.335	–	–	–	–	–	–
	Moderate	5.33 ± 4.382		-0.013	-0.017	0.986	–	–	–
	Severe	6.97 ± 8.549		-1.657	-1.569	0.118	-1.644	-1.490	0.137
Interpersonal Relationship	Mild	6.01 ± 5.375	2.255	–	–	–	–	–	–
	Moderate	5.4 ± 4.658		0.603	0.814	0.416	–	–	–
	Severe	7.73 ± 6.929		-1.719	-1.640	0.102	-2.323	-2.123	0.035
Depression	Mild	7.73 ± 7.334	2.756	–	–	–	–	–	–
	Moderate	7.03 ± 6.576		0.701	0.670	0.504	–	–	–
	Severe	10.64 ± 10.571		-2.902	-1.959	0.051	-3.603	-2.331	0.021
Total scores of SCL-90	Mild	49.91 ± 40.091	2.603	–	–	–	–	–	–
	Moderate	45.92 ± 35.498		3.993	0.692	0.49	–	–	–
	Severe	65.27 ± 60.522		-15.359	-1.881	0.061	-19.351	-2.270	0.024
Trait Anxiety	Mild	41 ± 10.227	1.206	–	–	–	–	–	–
	Moderate	40.78 ± 10.014		0.225	0.160	0.873	–	–	–
	Severe	43.85 ± 10.515		-2.848	-1.432	0.153	-3.073	-1.480	0.14
State Anxiety	Mild	40.01 ± 10.005	2.19	–	–	–	–	–	–
	Moderate	40.51 ± 9.457		-0.498	-0.367	0.714	–	–	–
	Severe	44 ± 10.198		-3.992	-2.078	0.039	-3.494	-1.743	0.083
Total scores of STAI	Mild	80.77 ± 19.926	1.792	–	–	–	–	–	–
	Moderate	81.28 ± 18.803		-0.507	-0.187	0.851	–	–	–
	Severe	87.88 ± 20.409		-7.105	-1.857	0.064	-6.598	-1.652	0.1

### 3.5 Relationships between SCL-90, STAI scores and TT

**Figure 1:**
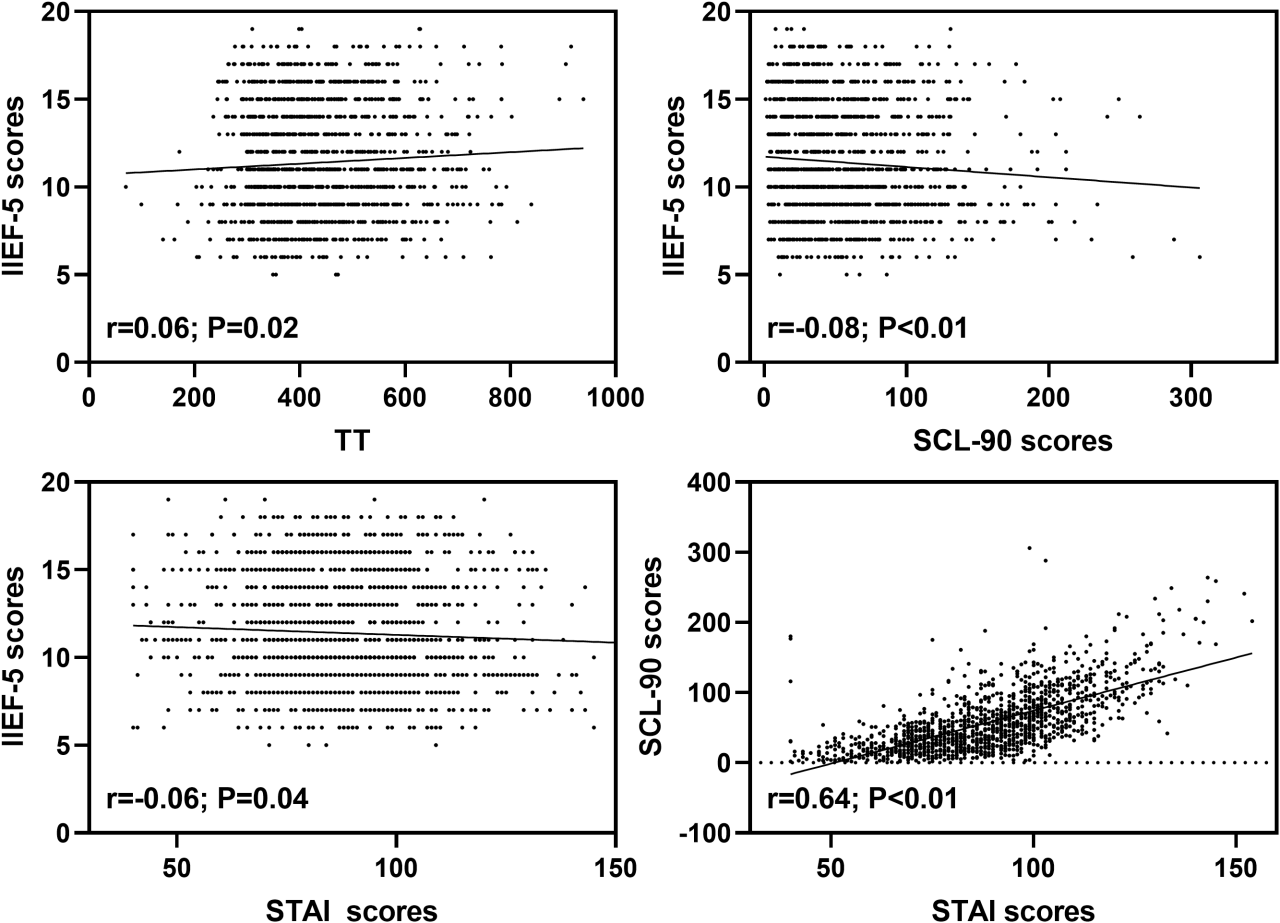
Relationships between SCL-90, STAI scores and TT.

The level of TT was positively related to IIEF-5 scores of ED patients (*r* = 0.06; *P* = 0.02). In addition, both SCL-90 (*r* = -0.08; *P* < 0.01) and STAI (*r* = -0.06; *P* = 0.04) scores were negatively associated with IIEF-5 scores of ED patients. Moreover, STAI scores were positively related to SCL-90 scores of ED patients (*r* = 0.64; *P* < 0.01) (**Figure 1**).

## 4 Discussion

ED, as a common male disease, seriously affects the sexual quality of life of patients and their sexual partners (32). It has been found that the incidence rate of ED is gradually increasing with age, and main patients are over 40 years old (33). However, a recent population-based study has demonstrated that with the change of people’s living habits, the ED population is becoming younger (34). Consequently, elucidating the incidence trends in younger populations and conducting age-stratified comparative analyses of disease manifestations could enable early intervention and precision medicine approaches.

TT is a key factor in regulating male sexual response, and low TT level is related to low libido in male (35, 36). Meanwhile, testosterone-deficient metabolic syndrome is significantly associated with severe ED (12). This study revealed lower TT levels in patients with severe ED compared to those with mild ED. Low TT level is considered as a sentinel manifestation of metabolic syndrome, and is also closely related to penile vascular injury, diabetes and other causes of ED (37). In this study, there was a significant correlation between TT and age: TT level showed a decline with age in ED patients. However, in patients aged 20–30 years with ED, there was no significant difference in TT levels among patients with ED of different levels of ED severity. Only older patients with higher ED severity showed significantly lower TT levels. This paradoxical finding, which contradicts previous studies reporting increasing ED prevalence among younger populations, suggests that non-hormonal etiological factors may play a more predominant role in ED pathogenesis in young patients compared to older individuals. TT level, which gradually decreases with age, may cause the patient’s sexual desire to decline, thus reducing their attention to sexual life. In addition, the decrease of TT level and the progress of TT-related comorbidities make the ED severity of elderly patients higher. While the precise pathogenesis remains to be elucidated, one plausible explanation is that as low TT level does not exist for a long time in young patients, it may not cause low testosterone related comorbidities (38). Although isolated low TT factor may not manifest as overt ED symptoms, routine testosterone monitoring remains clinically warranted in elderly patients, given the well-established association between hypogonadism and multiple age-related comorbidities.

It is undeniable that psychological factors play a key role in the occurrence and development of ED (39–41). Accumulated studies have established that a bidirectional relationship between psychological distress and ED: chronic anxiety and depression serves as a risk factor for ED pathogenesis, and ED caused by diverse etiology is also easy to cause psychological disorders of various severity (42–44). Our results demonstrate that the psychological burden of ED manifests acutely in younger populations, exhibiting consistent psychological problems regardless of ED severity. An age-stratified study (N = 948) revealed that 85.2% of ED cases among young males (<40 years) were primarily attributable to psychological etiology (45). Failed sexual behavior may trigger anxiety, decreased self-confidence, and subsequent avoidance behavior, thereby significantly elevating the likelihood of sexual behavior failure. Especially for adolescents with limited sexual experience demonstrate greater vulnerability to the negative consequences of sexual performance difficulties compared to middle-aged and elderly individuals (46). These psychological disturbances primarily manifest as persistent self-doubt, which significantly compromise subsequent sexual performance. This bidirectional interaction forms a self-perpetuating pathogenic cycle.

Our findings demonstrate an age-dependent attenuation in the correlation between psychological comorbidities and ED severity. Specifically, while patients aged 31–40 years with mild ED exhibited significant anxiety and depression symptoms, this psychological-disturbance gradient diminished progressively with advancing age. Only severe ED cases demonstrated pronounced psychological comorbidities in older age groups (≥41 years). There was a certain correlation between the decline of sexual life frequency and low life satisfaction, especially in young people and people over 60 years old (47, 48). However, for the elderly, sexual intercourse is not the key to maintaining sexual activity (49). Advanced-age patients typically demonstrate lower sexual activity frequency compared to younger populations, which may be attributed to age-related testosterone decline. Consequently, mild ED symptoms often fail to elicit significant anxiety in elderly patients.

Collectively, our findings delineate that psychological factor emerge as the primary etiological contributors in younger cohorts, whereas age-related hypogonadism and metabolic syndrome progressively assume dominant roles in older populations. While this study provides novel insights into age-related etiological differences in ED, several limitations warrant acknowledgment: (1) owing to the clinical recruitment process, the distribution of participants across age strata deviated from the anticipated equal allocation; (2) the reliance on self-reported psychological assessments could introduce recall bias. Future studies should address these gaps through broader inclusion criteria, and clinician-administered psychometric tools.

## 5 Conclusion

Endocrine and psychological factors exert distinct influences on ED across different ages. Young patients exhibit a heightened vulnerability to psychological distress, including anxiety and depression, despite generally presenting with milder ED severity. This phenomenon stems from the heightened sociosexual demands associated with maintaining intimate partnerships. Consequently, targeted psychological interventions should be prioritized in clinical management to mitigate both the symptoms of ED and their associated psychosocial sequelae in this population. The prevalence and severity of ED escalate with advancing age, due to age-related declines in TT levels and the progressively increasing incidence of related comorbidities. Comprehensive management of ED in elderly patients necessitates a multifactorial etiological analysis and the implementation of individualized, multidisciplinary treatment strategies.

## Data Availability

The raw data supporting the conclusions of this article will be made available by the authors, without undue reservation.
